# Genomic Data of *Acaciella* Nodule Ensifer mexicanus ITTG R7^T^

**DOI:** 10.1128/MRA.01251-20

**Published:** 2021-04-01

**Authors:** Reiner Rincón-Rosales, Marco A. Rogel, Gabriela Guerrero, Clara Ivette Rincón-Molina, Aline López-López, Luis Alberto Manzano-Gómez, Víctor Manuel Ruíz-Valdiviezo, Esperanza Martínez-Romero

**Affiliations:** a Tecnológico Nacional de México/I.T. de Tuxtla Gutiérrez, Tuxtla Gutiérrez, Chiapas, Mexico; b Centro de Ciencias Genómicas, Universidad Nacional Autónoma de México, Cuernavaca, Morelos, Mexico; c Departamento de Investigación y Desarrollo, 3R Biotec SA de CV, Tuxtla Gutiérrez, Chiapas, Mexico; University of Arizona

## Abstract

We report the complete genome sequence of Ensifer mexicanus ITTG R7^T^, a nitrogen-fixing bacterium isolated from nodules of Acaciella angustissima plants growing naturally in Chiapas, Mexico. The genome is distributed in four replicons comprising one 4.31-Mbp chromosome, one 1,933-Kb chromid, and two plasmids of 436 and 455 Kb.

## ANNOUNCEMENT

Ensifer mexicanus ITTG R7^T^ was isolated from Acaciella angustissima, which is distributed from the United States to Costa Rica (1). ITTG R7^T^ forms nodules and fixes nitrogen in Acacia cochliacantha, Phaseolus vulgaris, and Leucaena leucocephala (1). High levels of nitrogenase activity were obtained with *E. mexicanus* ITTG R7^T^ inoculated in Phaseolus vulgaris (2).

The strain ITTG R7^T^ was obtained from *Acaciella angustissima* nodules on peptone yeast extract (PY) plates and purified by streaking twice; it was stored at −70°C on the same medium containing 30% (vol/vol) glycerol. ITTG R7^T^ was grown in liquid PY medium, and DNA was obtained with the DNA isolation kit for cells and tissues from Roche. The genome was sequenced using the Pacific Biosciences (PacBio) RS II single-molecule real-time (SMRT) sequencing platform. Two SMRT cells of a 15- to 20-kb insert library were sequenced; 217,833 reads with an average read length of 14,296 bp were used for the *de novo* genome assembly using the program RS_HGAP_Assembly.3 in SMRT Portal Analysis v2.3.0. (3), where the PreAssembler Filter v1 is the function that cleans the reading considering different parameters. The whole genome was assembled with a coverage of 234×. Genome annotation was performed using the NCBI Prokaryotic Genome Annotation Pipeline (4). Clusters of orthologous groups (COGs) were obtained using Conserved Domain Search service (CD-Search) from the NCBI database, and hits were accepted with an E value of 1e^−10^. The rRNA operons were identified with RNAmmer (5). The PHAST program was used for prophage identification (6). Default parameters were used for all software.

The 7,141,863-bp genome sequence of ITTG R7^T^ is distributed in one chromosome of 4.31 Mbp and 3 replicons of 436 (pA), 455 (pB), and 1,933 (pC) kb with 62, 60, 59, and 62% GC contents, respectively. All replicons are circular, since an overlap of approximately 10 kb was found in each of them. The COG assignments to the open reading frame (ORF) were 3,494, 318, 279, and 1,677 for the chromosome, pA, pB, and pC, respectively, with 1,025, 145, 160, and 350 unassigned ORFs, respectively. pSym is the replicon that has the greatest percentage of genes (36.4%) without a COG assignment. A large number of genes involved in carbohydrate transport and metabolism were found in pC with 274 genes, in contrast to the chromosome, which has 221. Genes for secretion system types I, II, III, and IV were found distributed over the genome. Three ribosomal operons and three incomplete prophage regions were identified in the chromosome. Both pA and pB plasmids could be conjugative plasmids since they have the *tra* and *trb* genes. *nod* and *nif* genes were found in the 455-kb (pB) replicon, which was thus identified as the symbiosis plasmid (pSym) and had the lowest GC content, as has been reported for most of the pSyms of other rhizobia. The presence of *nodU* could promote *Leucaena leucocephala* nodulation (7). pSym has reiterated genes; two *nodA* genes were found that could explain its wide host range (1). In addition, two *nifX* and two *nifN* genes were found as well, which are involved in the biosynthesis of the iron-molybdenum cofactor (FeMo-Co) required for nitrogenase (8). Three clusters of *phn* genes that confer the ability to degrade phosphonates (organophosphorus compounds) (9) were found in the genome, one in the chromosome, one in the pC, and the last one in the pSym with 5, 7, and 8 genes, respectively. *hyp* genes related to the generation of metalloenzymes, such as nitrogenases, ureases, or hydrogenases, were found in pSym; some proteins encoded by these genes, such as the nickel-binding HypA, have been linked to virulence in human pathogens such as Helicobacter pylori and Escherichia coli (10). Genes for biotin biosynthesis, such as *birA* (biotin-[acetyl-coenzyme A {CoA}-carboxylase] ligase), a regulator of biotin biosynthesis in *Bacillus* (11), *bioC*, involved in the synthesis of pimeloyl-CoA biotin precursor, *bioADFB*, which converts pimeloyl-CoA to biotin, and three biotin transporters (*bioY*) (12), and genes for cobalamin biosynthesis were found. Thiamin biosynthesis genes *thiP* and *thiQ* were in the chromosome, while *thiS*, *thiO*, and *thiC* were in the pC chromid (13). pC was considered a chromid based on the criteria of GC content equal to that of the chromosome and the content of essential genes (13).

### Data availability.

The whole-genome nucleotide sequence of Ensifer mexicanus ITTG R7^T^ has been deposited in GenBank under the accession numbers CP041238, CP041239, CP041240, and CP041241 and SRA numbers SRR13240057 and SRR13240058.
